# Assessment of left ventricular function in patients with diabetic nephropathy using two-dimensional speckle-tracking echocardiography

**DOI:** 10.3389/fendo.2025.1547078

**Published:** 2025-06-16

**Authors:** Xiunan Zhang, Cuiping Jiang, Zhibin Ye, Yinjia Zhang, Xiaoli Zhang

**Affiliations:** ^1^ Department of Nephrology, Huadong Hospital, Fudan University, Shanghai, China; ^2^ Department of Endocrinology, Huadong Hospital, Fudan University, Shanghai, China; ^3^ Department of Ultrasound, Huadong Hospital, Fudan University, Shanghai, China

**Keywords:** diabetic nephropathy, albuminuria, subclinical left ventricular systolic dysfunction, GLS (global longitudinal strain), two-dimensional speckle-tracking echocardiography

## Abstract

**Objective:**

To assess cardiac function using two-dimensional speckle-tracking echocardiography (2D-STI) in diabetic nephropathy (DN) patients and investigate the relationship between albuminuria and early cardiac systolic and diastolic dysfunction, along with associated risk factors based on clinical indicators.

**Methods:**

A total of 75 patients with DN, 100 patients with diabetes mellitus (DM), and 37 healthy controls were recruited. Clinical data were collected, and conventional echocardiography as well as 2D-STI were performed on all participants.

**Results:**

2D-STI findings revealed a significant increased occurrence rate of subclinical left ventricular systolic dysfunction [global longitudinal strain values (GLS) <18%], among diabetic patients compared to healthy controls. Furthermore, the proportion of GLS<18% occurrence was higher in the DN group compared to the DM group (p<0.001) and especially higher in the massive albuminuria group than that in the microalbuminuria group (*p*<0.001). The results demonstrated that albuminuria, eGFR<60 ml/min/1.73 m^2^, and total cholesterol were identified as significant risk factors for the development of subclinical left ventricular systolic insufficiency in diabetic patients. However, when considering only patients with DN and adjusting for covariates, it was found that only total cholesterol remained statistically significant (*p*< 0.05).

**Conclusion:**

The higher cholesterol levels in patients with DN are associated with a greater risk of subclinical left ventricular systolic dysfunction reflected by a decrease in GLS assayed with 2D-STI.

**Critical relevance statement:**

GLS measured by 2D-STI combined with clinical indexes to evaluate and predict subclinical left ventricular systolic function in patients with DM, providing reference for early prevention and treatment of cardiac dysfunction in patients with DN.

## Highlights

- Subclinical left ventricular systolic dysfunction assessed by GLS in DM.
- Cholesterol was the risk factor for subclinical left ventricular systolic insufficiency in DN.
- Providing early detection measures of left ventricular systolic dysfunction in DN.

## Introduction

According to statistics, the global prevalence of DM is 10.5% in 2021, and this figure is expected to rise to 12.2% in 2045 (1). With the adoption of a westernized lifestyle and an aging population, the prevalence of DM in China has experienced a rapid surge from 0.67% in 1980 to 10.4% in 2013, marking a sixteen-fold increase over a span of thirty years (2). Furthermore, based on a survey conducted in 2010, the prevalence of prediabetes in China was found to be at approximately 50.1%, indicating that around half a billion Chinese adults are affected by prediabetic conditions. As such, China currently harbors the largest diabetic population globally with no signs of abating (3).

DN is one of the microvascular complications of diabetes and occurs in about 40% of diabetic patients. The most significant cardiovascular complication in diabetes is premature coronary atherosclerosis leading to ischemic heart disease. Another diabetic cardiovascular complication, diabetic cardiomyopathy (DCM), has garnered increased attention in recent years. DCM is now recognized as being an alteration occurring within myocardial structure and function specifically related to DM independent from other established cardiovascular risk factors like coronary artery disease, major valvular disease, or hypertension (4). It has been suggested that altered cardiac function observed in DCM initially manifests as impaired diastolic function followed by impaired systolic function during later stages (5).

Patients afflicted with DN face an exceptionally high risk for experiencing cardiovascular events which remain the primary cause of mortality amongst this group. This heightened risk can only be partially attributed to DM itself along with other comorbidities commonly associated with DM including hypertension and obesity. Identifying novel risk factors that underpin the association between DN and cardiovascular disease (CVD) is crucial for risk stratification, personalized treatment and identification of new therapeutic targets (6). Currently, both albuminuria and reduced GFR are acknowledged as independent risk factors and predictors of CVD development (7–9).

Presently, domestic and international expert consensus and guidelines consistently advocate for albuminuria as a pivotal criterion for DN diagnosis and prognostic evaluation. Studies have demonstrated that albuminuria is independently associated with left ventricular systolic and diastolic hypofunction, which may elucidate the close correlation between proteinuria and the heightened incidence of CVD in DN patients (10). Some studies have indicated an association between proteinuria levels and increased occurrence of left ventricular hypertrophy (LVH) (11–13) and myocardial fibrosis (14) in DN patients. Therefore, the role of albuminuria in the structural and functional changes of the heart in DN patients should not be underestimated.

Echocardiography, being a non-invasive imaging technique, possesses unique advantages in assessing cardiac functional changes. 2D-STI, a novel ultrasound imaging technique designed to objectively quantify myocardial function while providing information about myocardial strain, can identify discrete changes that conventional echocardiography fails to detect while offering additional prognostic insights (17).

In this study, 2D-STI was utilized to assess the cardiac function of patients with DN. This method was combined with clinical parameters to investigate the correlation between albuminuria and the development of cardiac systolic and diastolic dysfunction. The primary objective was to detect early changes in cardiac function in DN patients and implement prompt intervention and treatment. This approach may serve as a foundation for evaluating clinical outcomes and predicting prognosis.

## Methods

### Study population

75 patients with DN and 100 patients with non-nephropathic DM clinically diagnosed at the Huadong Hospital Affiliated to Fudan University from November 2021 to March 2023 were selected for the study. Inclusion and exclusion criteria for the DN group: patients clinically diagnosed with DN in our hospital (for diagnostic criteria, see 2022 KDIGO Clinical Practice Guidelines for the Management of Diabetes Mellitus in Chronic Kidney Disease) (15) were eligible, adults and ≤ 80 years of age, and were eligible if they had no known cardiac disease. Exclusion criteria included (1) end-stage renal disease with maintenance hemodialysis or peritoneal dialysis, or renal transplantation; (2) comorbid nondiabetic renal disease, including other primary and secondary glomerular or systemic diseases; hereditary renal disease; normoalbuminuric DN; and (3) comorbid known cardiac disease including heart failure; coronary artery disease (CAD). CAD: previous stable angina, myocardial infarction, previous percutaneous coronary intervention or after coronary artery bypass grafting; atrial flutter or atrial fibrillation, left or right bundle branch block, congenital heart disease, implantation of a pacemaker or implantable cardioverter-defibrillator; and congenital heart disease; (4) LVEF < 50%; and (5) malignant tumor. Inclusion and exclusion criteria for the DM alone group: patients who were clinically diagnosed with DM by our hospital (see ADA2021 guidelines for diagnostic criteria) (16), all of whom were adults and ≤80 years of age, and were eligible if they had no known heart disease or renal disease. Thirty-seven persons who came to our hospital for a physical examination during the same period and were excluded from having heart disease by taking a medical history and performing an echocardiogram were used as healthy controls.

The study complied with ethical guidelines and was approved by the Ethics Review Committee of the Huadong Hospital Affiliated to Fudan University, and all participants signed a written informed consent.

### Collection of general data and biochemical indicators

Age, sex, systolic blood pressure, diastolic blood pressure, height and weight were collected from patients with DN, non-nephropathic DM and healthy controls, and the body mass index (BMI) and body surface area (BSA) of the subjects were calculated according to the formula. Blood counts (hemoglobin, erythrocyte distribution width), C-reactive protein (CRP), urinary albumin-to-creatinine ratio (UACR), renal function (serum creatinine, urea nitrogen), serum albumin, and lipids (HDL, LDL, triglycerides, cholesterol), glucose metabolism (fasting glucose, glycosylated hemoglobin) and other related parameters.

### Acquisition of routine echocardiographic parameters

A GE Vivid E95 diagnostic ultrasound machine was used. Before echocardiography, blood pressure and electrocardiogram at rest were recorded in supine position in all patients. Routine echocardiography was performed first to exclude subjects with any previously undetected structural heart disease, including cardiomyopathy, valvular disease, or pericardial disease. Routine measurements of left atrial anteroposterior diameter (LAD), left ventricular end-diastolic internal diameter (LVEDd), left ventricular end-systolic internal diameter (LVESd), left ventricular ejection fraction (LVEF), interventricular septal thickness (IVST), and left ventricular posterior wall thickness (LVPWT) were performed. Spectral and tissue wave Doppler examinations were performed in apical 4-chamber cardiac views to obtain flow velocities in early (E) and late (A) mitral orifice diastole. Mean values of mitral annular septum and left ventricular lateral wall early diastolic blood flow velocities (e’) were determined from four-chamber cardiac views of the lateral region of the mitral annulus, and E/A and E/e’ were calculated. And left ventricular mass index (LVMI) was calculated according to the formula.

### Acquisition of 2D-STI parameters

After the routine parameter acquisition was completed, the M5S 2D probe was utilized to acquire three consecutive cardiac cycle images in apical two-chamber, apical three-chamber, and apical four-chamber views, respectively. Parameters were acquired in such a way that the myocardium between the epicardial and endocardial contour lines was used as the tracking range, and all the myocardium of the left ventricle was delineated into the speckle tracking range. After the endocardial rim was automatically outlined, the width of the region of interest was manually adjusted to equal the myocardial thickness to ensure that all LV myocardial segments were included and accurately tracked throughout the cardiac cycle. Strain fractions were subsequently calculated for the 3 sections. The global longitudinal strain value (GLS) was determined as the average peak strain of the 17 segments obtained from the 3 standardized views of the sections and expressed as an absolute value. The 17-segment model was used for the segmentation of LV myocardium in this study: the LV myocardium was divided into 3 myocardial rings (apical, intermediate, and basal), and in short-axis views, the intermediate and basal rings were each divided into 6 equal 60° segments, the apical ring was divided into 4 equal 90° segments, and the portion of the myocardium that extends beyond the end of the cardiac chambers was defined as the apical cap; this resulted in 17 segments. Subclinical LV systolic insufficiency was regularly defined in this study as LVEF ≥50% and GLS <18% (29).

All of the above echocardiographic images were acquired and analyzed by the same physician with more than 10 years of experience in ultrasonography.

### Statistical analysis

SPSS 26.0 software was used for statistical analysis. Measurement data conforming to normal distribution were expressed as mean ± standard deviation (SD), *t*-test was used for comparison between two groups, and one-way ANOVA was used for comparison between multiple groups; non-normally distributed measurement data were expressed as median (interquartile range), Mann-Whitney U test was used for comparison between two groups, and Kruskal-Wallis test was used for comparison between multiple groups. Count data were expressed as frequency (percentage), and the χ^2^ test was used. Logistic regression model was used to analyze the relationship between multiple factors and the occurrence of subclinical left ventricular systolic insufficiency and diastolic insufficiency, and the relative risk ratios (ORs) and 95% confidence intervals (CIs) were estimated, and p<0.05 was considered indicative of significance. Processing of missing data: This study intends to adopt multiple methods to handle missing data: Directly delete variables with missing values >30%; For continuous variables of normal distribution with fewer missing values, the mean value is used for filling, and for continuous variables of non-normal distribution, the median is used for filling.

## Results

### Baseline information

A total of 75 patients with DN and 100 patients with non-nephropathic DM were included in this study. Microalbuminuria accounted for 42 patients and macroalbuminuria accounted for 33 patients with DN. In addition, 37 healthy volunteers were included as controls. Baseline statistics of all patients are shown in **Table 1A** and **Table 2A**.

**Table 1A:** Characteristics of the clinical data of each group.

Variables	DN (n=75)	DM (n=100)	Control (n=37)	p
Sex (male%)	50 (66.7%)^①②^	46 (46.0%)^①^	17 (45.9%)	0.016
Age (years)	63.00 (57.00, 69.00)	62.00 (58.00, 68.75)	64.00 (50.50, 69.50)	0.910
BMI (kg/m^2^)	24.56 ± 2.87	23.78 ± 3.09	23.62 ± 2.60	0.146
Duration of diabetes (years)	10.00 (6.00, 20.00)	9.50 (2.00, 17.00)	NA	0.037
Smoking history	17 (22.7%)	25 (25.0%)	6 (16.2%)	0.552
Systolic blood pressure (mmHg)	132.72 ± 12.07^①②^	124.02 ± 9.81	121.81 ± 9.99	0.000
Diastolic blood pressure (mmHg)	81.41 ± 8.21^①②^	75.93 ± 8.63	77.05 ± 7.38	0.000
Hemoglobin (g/L)	122.43 ± 25.63^①②^	134.04 ± 13.34	134.89 ± 18.21	0.004
Erythrocyte distribution width (%)	12.90 (12.40, 13.50)	12.90 (12.50, 13.28)	12.90 (12.55, 13.20)	0.982
CRp(mg/L)	2.14 (0.84, 4.78)^②^	1.20 (0.67, 2.69)^①^	2.72 (1.01, 4.74)	0.002
Serum creatinine (umol/L)	90.90 (61.00, 147.90)^①②^	62.65 (53.60, 77.80)	71.20 (56.15, 80.80)	0.000
Urea nitrogen (mmol/L)	7.80 (5.00, 11.0)^①②^	5.20 (4.30, 6.30)	5.10 (4.20, 5.98)	0.000
eGFR (ml/min/1.73m^2^)	69.00 (34.00, 94.00)^①②^	94.00 (86.00, 101.75)	90.00 (78.50, 103.50)	0.000
Triglycerides (mmol/L)	1.33 (0.97, 2.17)	1.46 (1.13, 2.01)	1.15 (0.82, 1.74)	0.131
Total cholesterol (mmol/L)	4.18 (3.43, 5.05)^①^	4.34 (3.72, 5.04)^①^	4.98 (4.63, 5.59)	0.004
HDL (mmol/L)	1.15 (0.95, 1.40)^①^	1.19 (1.03, 1.39)^①^	1.36 (1.22, 1.59)	0.001
LDL (mmol/L)	2.28 (1.62, 2.92)^①^	2.49 (1.99, 2.98)^①^	3.03 (2.64, 3.41)	0.001
Serum albumin (g/L)	40.00 (34.30, 42.80)^①②^	41.90 (40.10, 44.38)	41.80 (39.75, 44.35)	0.000
Glycated hemoglobin (%)	7.30 (6.40, 9.53)	8.15 (6.83, 10.38)	NA	0.036
Fasting blood glucose (mmol/L)	6.30 (4.90, 8.20)^①^	6.20 (5.33, 7.18)^①^	4.80 (4.60, 5.30)	0.000

Depending on the distribution, continuous variables are expressed as mean ± SD or median (interquartile range), and categorical variables are expressed as frequency (percentage).

① p<0.05 compared with the control group.

② p<0.05 compared with the diabetes-only group.

**Table 1B:** Characteristics of echocardiographic indices in each group.

Routine echocardiogram	DN (n=75)	DM (n=100)	Control (n=37)	p
LAD (mm)	37.61 ± 4.96^①②^	35.28 ± 3.77	34.27 ± 4.55	0.000
LVEDd (mm)	45.22 ± 4.49	43.71 ± 3.60	44.32 ± 3.16	0.066
LVESd (mm)	28.26 ± 3.90	27.36 ± 2.96	28.00 ± 3.15	0.203
IVST (mm)	10.00 (9.00, 10.00)^①②^	9.00 (8.00, 10.00)	9.00 (8.00, 9.50)	0.005
LVPWT (mm)	9.00 (9.00, 10.00)^①②^	9.00 (8.00, 9.00)	9.00 (8.00, 9.00)	0.022
LVMI (g/m2)	83.36 ± 21.16^①②^	74.36 ± 14.72	75.92 ± 14.66	0.031
LVEF (%)	64.50 (61.00, 68.00)	66.00 (62.00, 68.00)	66.00 (61.00, 68.00)	0.406
SV (ml)	54.00 (45.75, 67.50)	50.00 (45.00, 59.00)	54.00 (44.00, 63.50)	0.130
LVEDV (ml)	86.50 (72.00, 106.25)	77.00 (68.00, 92.00)	84.00 (68.50, 98.50)	0.086
HR(beats/min)	75.47 ± 11.27	73.84 ± 11.30	70.97 ± 9.79	0.131
E	70.00 (57.75, 82.25)	67.00 (58.00, 76.00)	69.00 (52.50, 82.00)	0.648
A	91.50 (77.75, 105.25)^①②^	87.00 (75.00, 95.00)	79.00 (67.50, 96.00)	0.007
E/A	0.74 (0.63, 0.89)	0.77 (0.66, 0.88)	0.80 (0.71, 0.94)	0.123
IVRT (ms)	86.99 ± 18.50	84.11 ± 13.81	84.03 ± 16.62	0.461
E/e’	9.15 (8.10, 11.65)^①②^	8.60 (7.40, 10.00)	8.50 (6.23, 9.65)	0.008
Diastolic insufficiency (E/e’ >14)	10 (13.3%)	5 (5.0%)	1 (2.7%)	0.054
2D-STI				
GLS (%)	16.00 (14.30, 17.50)^①②^	17.80 (16.70, 19.10)^①^	19.80 (18.05, 20.65)	0.000
Subclinical left ventricular systolic dysfunction (GLS <18%)	61 (81.3%)^①②^	54 (54.0%)^①^	7 (18.9%)	0.000

Depending on the distribution, continuous variables are expressed as mean ± SD or median (interquartile range), and categorical variables are expressed as frequency (percentage).

① p<0.05 compared with the control group.

② p<0.05 compared with the diabetes-only group.

**Table 2A:** Comparison of general information of all diabetic patients.

Variables	Normal albuminuria (n=100)	Microalbuminuria (n=42)	Macroalbuminuria (n=33)	p
Sex (male%)	46 (46.0%)	25 (59.5%)①	25 (75.8%)①②	0.009
Age (years)	62.00 (58.00, 68.75)	60.50 (52.75, 68.25)	65.00 (58.50, 70.50)	0.365
BMI (kg/m2)	23.78 ± 3.09	24.68 ± 3.14	24.40 ± 2.51	0.22
Duration of diabetes (years)	9.50 (2.00, 17.00)	10.00 (1.00, 18.50)	14.00 (10.00, 20.00)①②	0.013
Smoking history	25 (25.0%)	8 (19.0%)	9 (27.3%)	0.666
Systolic blood pressure (mmHg)	124.02 ± 9.81	130.02 ± 11.14①	136.15 ± 12.50①②	0.000
Diastolic blood pressure (mmHg)	75.93 ± 8.63	81.60 ± 9.00①	81.18 ± 7.22①	0.000
Hemoglobin (g/L)	134.04 ± 13.34	132.64 ± 20.31	109.42 ± 26.07①②	0.000
Erythrocyte distribution width (%)	12.90 (12.50, 13.28)	12.80 (12.38, 13.40)	12.90 (12.45, 13.80)	0.504
CRp (mg/L)	1.20 (0.67, 2.69)	1.72 (0.80, 3.97)	2.91 (1.20, 4.90)①	0.016
Serum creatinine (umol/L)	62.65 (53.60, 77.80)	68.25 (55.38, 100.05)	147.90 (111.25, 327.25)①②	0.000
Urea nitrogen (mmol/L)	5.20 (4.30, 6.30)	5.40 (4.30, 8.65)	10.70 (8.00, 19.15)①②	0.000
eGFR (ml/min/1.73m^2^)	94.00 (86.00, 101.75)	93.00 (67.75, 105.00)	34.00 (18.50, 54.50)①②	0.000
Triglycerides (mmol/L)	1.46 (1.13, 2.01)	1.33 (0.99, 2.18)	1.32 (0.90, 2.21)	0.807
Total cholesterol (mmol/L)	4.34 (3.72, 5.04)	4.03 (3.55, 4.82)	4.50 (3.12, 5.34)	0.350
HDL (mmol/L)	1.19 (1.03, 1.39)	1.06 (0.86, 1.42)	1.22 (1.05, 1.36)	0.225
LDL (mmol/L)	2.49 (1.99, 2.98)	2.09 (1.65, 2.80)	2.51 (1.53, 3.15)	0.218
Serum albumin (g/L)	41.90 (40.10, 44.38)	41.70 (38.58, 43.53)	35.50 (30.80, 40.15)①②	0.000
Glycated hemoglobin (%)	8.15 (6.83, 10.38)	8.55 (6.80, 10.18)	6.75 (6.13, 8.35)①②	0.002
Fasting blood glucose (mmol/L)	6.20 (5.33, 7.18)	7.00 (5.65, 8.83)①	5.40 (4.35, 6.65)①②	0.001

① Compared with the normoalbuminuria group, p< 0.05.

② Compared with the microalbuminuria group, p< 0.05.

**Table 2B:** Comparison of echocardiographic indicesof all diabetic patients.

Routine echocardiogram	Normal albuminuria (n=100)	Microalbuminuria (n=42)	Macroalbuminuria (n=33)	p
LAD (mm)	35.28 ± 3.77	36.24 ± 4.33	39.41 ± 5.22①②	0.000
LVEDd (mm)	43.71 ± 3.60	44.55 ± 4.26	46.09 ± 4.70①	0.014
LVESd (mm)	27.36 ± 2.96	27.60 ± 3.95	29.13 ± 3.73①	0.037
IVST (mm)	9.00 (8.00, 10.00)	9.00 (8.00, 10.00)	10.00 (9.00, 11.00)①	0.004
LVPWT (mm)	9.00 (8.00, 9.00)	9.00 (8.00, 9.00)	9.00 (9.00, 10.00)①②	0.003
LVMI (g/m^2^)	74.36 ± 14.72	78.48 ± 19.63	89.78 ± 21.67①②	0.003
LVEF (%)	66.00 (62.00, 68.00)	65.00 (61.75, 68.25)	64.00 (61.00, 67.00)	0.269
SV (ml)	50.00 (45.00, 59.00)	52.50 (42.50, 64.00)	58.00 (47.75, 76.50)①	0.031
LVEDV (ml)	77.00 (68.00, 92.00)	78.50 (67.25, 101.25)	96.00 (75.25, 120.50)①②	0.011
HR(beats/min)	73.84 ± 11.30	75.95 ± 12.33	74.84 ± 9.86	0.59
E	67.00 (58.00, 76.00)	65.00 (56.50, 79.50)	75.00 (64.00, 93.00)	0.089
A	87.00 (75.00, 95.00)	88.50 (75.75, 98.25)	99.00 (84.25, 109.00)①②	0.008
E/A	0.77 (0.66, 0.88)	0.72 (0.63, 0.86)	0.76 (0.63, 0.91)	0.517
IVRT (ms)	84.11 ± 13.81	83.98 ± 16.18	90.94 ± 20.76	0.225
E/e’	8.60 (7.40, 10.00)	8.50 (7.65, 10.65)	10.60 (8.84, 13.42)①②	0.000
Diastolic insufficiency (E/e’ >14)	5 (5.0%)	1 (2.4%)	9 (27.3%)①②	0.000
2D-STI				
GLS (%)	17.80 (16.70, 19.10)	16.40 (14.30, 18.10)①	15.30 (13.60, 16.20)①②	0.000
Subclinical left ventricular systolic dysfunction (GLS <18%)	54 (54.0%)	30 (71.4%)	31 (93.9%)①②	0.000

① Compared with the normoalbuminuria group, p< 0.05.

② Compared with the microalbuminuria group, p< 0.05.

### Echocardiographic characteristics

The baseline echocardiographic characteristics are shown in **Table 1B** and **Table 2B**. Comparison of the cardiac ultrasound indices of all the included individuals revealed an increase in LAD, IVST, LVPWT, LVMI, A, E/e’, and LAEV in the DN group compared with the DM-only group (*p*< 0.05). Comparison of cardiac ultrasound indexes in all diabetic patients revealed that LAD, LVPWT, LVMI, LVEDV, A, E/e’ were significantly larger in the massive albuminuria group than in the microalbuminuria group, and LVEDd, LVESd, IVST, and SV were significantly increased compared with those of the normoalbuminuria group (*p*< 0.05).

**Figure 1:**
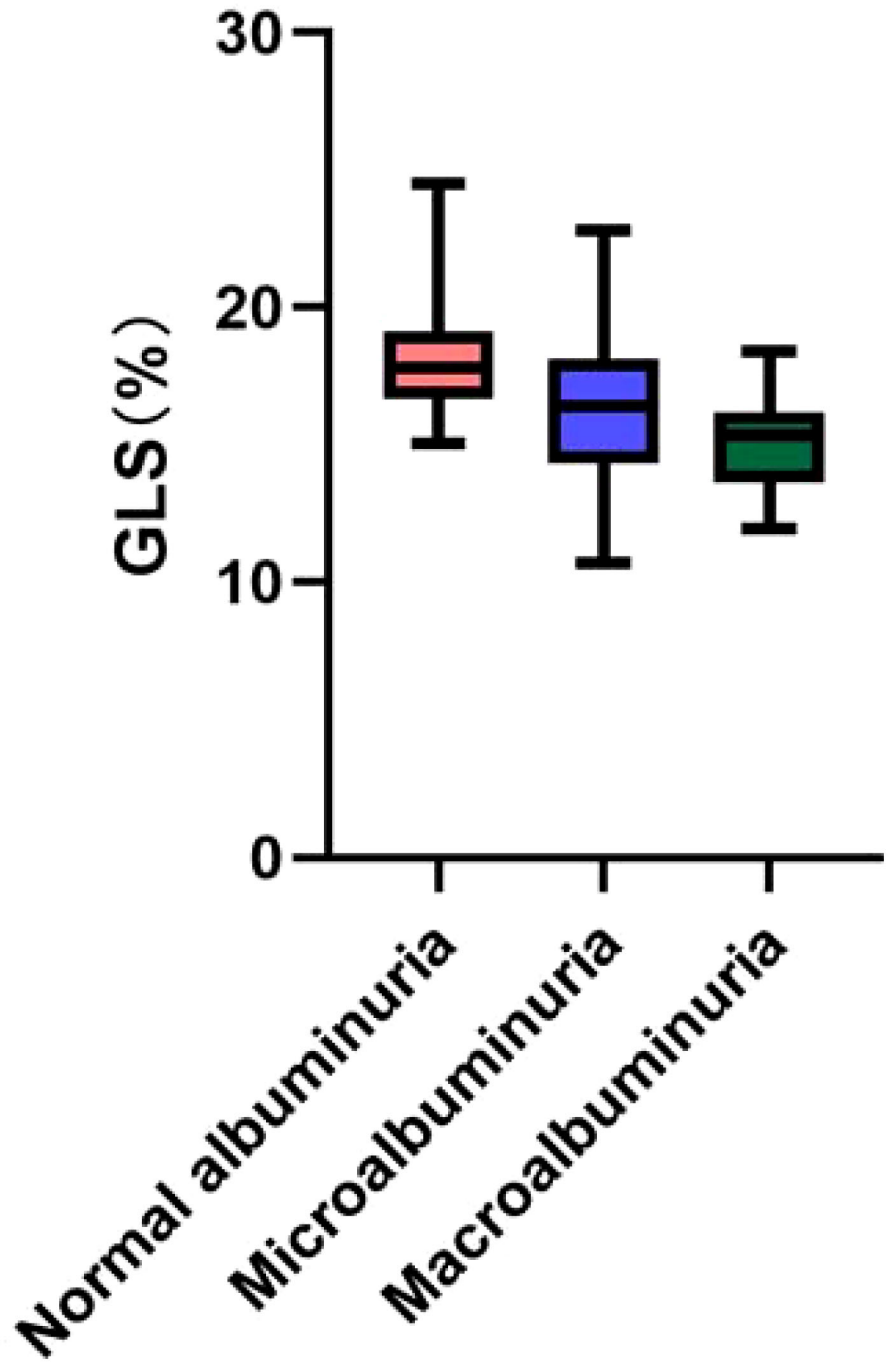
GLS levels in all diabetic patients.

2D-STI results showed a significant decrease in LV GLS and a significant increase in the proportion of occurrence of subclinical left ventricular systolic insufficiency (GLS <18%) in diabetic patients compared with healthy controls; GLS was further decreased in the DN group compared with the non-nephropathic DM group (16.00% vs. 17.80%, *p*< 0.001), and the proportion of occurrence of subclinical left ventricular systolic insufficiency (GLS < 18%) occurred in a significantly higher proportion (81.3% vs 54.0%) (*p*< 0.001). GLS gradually decreased with increasing albuminuria levels, and the proportion of subclinical left ventricular systolic insufficiency was significantly higher in the macroalbuminuria group (93.9%) than in the microalbuminuria group (71.4%) and the normoalbuminuria group (54.0%) (*p*< 0.001) (**Figure 1**). The percentage of occurrence of left ventricular diastolic insufficiency was significantly higher in the massive albuminuria group (27.3%) than in the microalbuminuria group (2.4%) and the normoalbuminuria group (5.0%) (*p*< 0.001) (**Tables 1B**, **2B**).

### Analysis of risk factors for developing subclinical left ventricular systolic insufficiency in DM patients

Multifactorial logistic regression analysis showed that albuminuria, eGFR <60 ml/min/1.73 m^2^, and total cholesterol were risk factors for the development of subclinical left ventricular systolic insufficiency in patients with DM, and the risk of subclinical left ventricular systolic insufficiency in patients with DN was 2.501 times higher than that of patients with albuminuria-negative DM alone. In contrast, when only DN patients were included and adjusted for age, sex, hypertension, duration of diabetes (in years), smoking history, obesity, triglycerides, cholesterol, proteinuria, and eGFR <60 ml/min/1.73 m^2^, it was found that only total cholesterol retained statistical significance (OR = 3.081, 95% CI 1.118-8.491, *p*< 0.05) (**Table 3**).

**Table 3:** Logistic regression analysis of the occurrence of subclinical left ventricular systolic dysfunction in patients with DM.

GLS<18%	Single factor		Multifactor model 1		Multifactor model 2	
	OR (95%CI)	P	OR (95%CI)	P	OR (95%CI)	P
Sex (male)	2.034 (1.080-3.832)	0.028	1.762 (0.745-4.167)	0.197	3.791 (0.681-21.109)	0.128
Age (years)	0.988 (0.956-1.020)	0.444	0.993 (0.952-1.035)	0.738	1.025 (0.945-1.111)	0.555
Duration of diabetes (years)	1.009 (0.974-1.046)	0.617	1.022 (0.979-1.068)	0.321	1.005 (0.912-1.107)	0.926
Smoking history	1.219 (0.579-2.567)	0.602	1.165 (0.426-3.182)	0.766	2.545 (0.213-30.372)	0.460
Obesity % (BMI ≥ 24)	1.700 (0.906-3.191)	0.099	1.596 (0.761-3.348)	0.216	0.287 (0.039-2.116)	0.221
Hypertension %	1.260 (0.658-2.412)	0.486	0.868 (0.390-1.933)	0.729	1.876 (0.222-15.864)	0.563
Albuminuria	3.712 (1.841-7.485)	0.000	2.501 (1.092-5.726)	0.030	3.623 (0.312-42.061)	0.303
eGFR <60ml/min/1.73m2	6.380 (2.149-18.942)	0.001	4.008 (1.180-13.612)	0.026	1.983 (0.211-18.607)	0.549
Triglycerides (mmol/L)	1.488 (1.028-2.153)	0.035	1.307 (0.870-1.966)	0.198	1.031 (0.443-2.396)	0.944
Total cholesterol (mmol/L)	1.382 (1.027-1.860)	0.033	1.552 (1.059-2.275)	0.024	3.081 (1.118-8.491)	0.030
HDL(mmol/L)	0.657 (0.256-1.691)	0.384				
LDL(mmol/L)	1.434 (0.986-2.087)	0.059				
Serum albumin (g/L)	0.952 (0.890-1.019)	0.157				
Glycated hemoglobin (%)	1.066 (0.929-1.223)	0.365				
Fasting blood glucose (mmol/L)	1.072 (0.936-1.227)	0.318				
Systolic blood pressure (mmHg)	1.018 (0.991-1.046)	0.195				
Diastolic blood pressure (mmHg)	1.013 (0.978-1.050)	0.474				
Hemoglobin (g/L)	0.992 (0.977-1.008)	0.343				
Erythrocyte distribution width (%)	0.903 (0.726-1.122)	0.355				
CRp(mg/L)	1.009 (0.980-1.039)	0.554				

Smoking history, obesity, triglycerides, cholesterol, proteinuria, and eGFR <60ml/min/1.73m^2^. Multifactorial model 1: adjusted for age, sex, hypertension, duration of diabetes (years); Multifactorial model 2: Only DN patients were included. Adjusted for age, sex, hypertension, duration of diabetes (years), smoking history, obesity, triglycerides, cholesterol, proteinuria, and eGFR <60ml/min/1.73m^2^.

## Discussion

The global rise in DM is attributed to the increasing rates of obesity, metabolic syndrome, and adoption of Westernized lifestyle. Consequently, there is a gradual increase in the prevalence of DN, a microvascular complication associated with DM (17). Data from a 2017 statistical report indicated that the age-standardized prevalence of DN was 15.48/1000 for men and 16.50/1000 for women worldwide (18). Ischemic cardiomyopathy is identified as the primary cause of mortality among diabetic patients. Furthermore, diabetic patients may experience microvascular disease and a decrease in cardiac capillary density in addition to coronary artery disease. Diabetic cardiomyopathy is a disease of the myocardium in diabetic patients that cannot be explained by the etiology of coronary atherosclerosis, hypertension, heart valve disease, and other heart diseases, which can be asymptomatic in the early stages and lead to heart failure in the late stages, and which occurs independently of other cardiovascular risk factors (19).

In this study, the incidence of subclinical left ventricular systolic insufficiency (GLS<18%) was found to be 65.7% (115/175) in DM patients, which was significantly higher than that in healthy controls. The occurrence of subclinical left ventricular systolic dysfunction in patients with DM has shown variability across various studies (20–22), potentially attributed to the inclusion of individuals with diverse clinical profiles, including varying durations of DM, severity of associated complications, and comorbidities. Limited research exists on the early cardiac functional alterations in DN patients. In our study, 75 patients with DN were included, and the 2D-STI results revealed that the incidence of subclinical left ventricular systolic insufficiency was 81.3% (61/75), which was significantly higher than that in patients with non-nephropathic DM, and with the increase in the degree of albuminuria, the GLS gradually decreased, which suggests that the incidence of subclinical left ventricular systolic insufficiency is gradually increasing.

In order to investigate the relationship between changes in the structural function of the heart and proteinuria in patients with DN, in this study, patients with DM were divided into three groups according to the level of albuminuria. The results showed that as levels of albuminuria increased, there was a corresponding increase in LVEDd, LVESd, and LV hypertrophy indices (manifested as an increase in LVMI, LVPWT, and LVST). Liu et al. have also reported that the LVPWT and LVMI increased progressively from the normoalbuminuric group to the macroalbuminuric group, and that mitral E/A ratio was lower in the microalbuminuric and macroalbuminuric groups compared to the normoalbuminuric group (10). Katz et al. found significant increase in LVMI and LVPWT with increasing UACR (23).

Patients with DN are at very high risk of cardiovascular events due to diabetes itself as well as diabetic complications and comorbidities, and it is currently believed that variables such as hypertension, hyperlipidemia, and obesity are potential cardiovascular risk factors in diabetic patients, but they do not fully explain the higher cardiovascular morbidity in patients with DN. The identification of new risk factors that support the association between DN and CVD is essential for risk stratification, individualization of treatment, and identification of new therapeutic targets (6, 24). This study aimed to investigate the risk factors associated with subclinical left ventricular systolic insufficiency in patients with DM. The results of multivariate Logistic regression analysis showed that proteinuria was an independent risk factor for subclinical left ventricular systolic insufficiency, and the risk of subclinical left ventricular systolic insufficiency in the DN group was 2.501 times higher than that in the DM group (normoalbuminuric group). Previous research has concentrated on the association between proteinuria and left ventricular function in individuals with DM. For instance, Katz et al. (23) found that log-transformed UACR was significantly negatively correlated with GLS, and that the negative correlation between the absolute values of UACR and GLS persisted after adjustment for DM, eGFR, and CAD. When only DN patients were included in Model 2 in this study, multifactorial logistic regression analysis suggested an increased risk of subclinical left ventricular systolic insufficiency in the massive albuminuria group compared with the microalbuminuria group, but the difference was not statistically significant. It was considered that the final results were influenced by the fact that some patients were already using ACEI/ARB analogs before the study. Additionally, the limited sample size of the massive proteinuria group included in this study could have contributed to the lack of statistically significant difference.

Several studies have indicated that, apart from albuminuria, decreased eGFR is an independent risk factor for CVD events in patients with DM (9). eGFR reduction may mediate the high incidence of CVD in patients with DN through nontraditional risk factors for CVD such as, hyperhomocysteinemia, elevated markers of oxidative stress and inflammation, anemia, and abnormalities of calcium and phosphorus metabolism (25). These factors have often not been adequately evaluated in previous research. Reduced eGFR by itself may be a risk factor for ventricular remodeling and progression of cardiac dysfunction, and may serve as a marker of CVD (26). Recent studies have also revealed that DN patients with reduced eGFR are less likely to receive cardioprotective drugs and therapies than patients with normal eGFR (27). Given the increased risk of CVD in patients with DN, the under-treatment among patients with decreased eGFR is of concern, which may be due to the fact that clinicians assessing the risk-benefit emphasize the risk of short-term side effects while ignoring the long-term benefit of reduced mortality. In our own study, an eGFR <60 ml/min/1.73 m2 was identified as a risk factor for the development of subclinical left ventricular systolic insufficiency in DM patients, although the statistical significance of eGFR was no longer evident when only DN patients were considered in the analysis.

In our study, it was observed that the prevalence of subclinical left ventricular systolic insufficiency in patients with DM and DN rose significantly in correlation with elevated total cholesterol levels. Previous clinical investigations have demonstrated that hyperlipidemia increases the risk of developing nonischemic heart failure and that lowering lipids reverses cardiac dysfunction. In addition to hyperlipidemia affecting cardiac function by promoting the development of atherosclerosis, it directly affects cardiac electrophysiological responses, which may be related to cardiac lipid deposition and the subsequent occurrence of systemic oxidative stress, inflammatory responses, and mitochondrial dysfunction (28).

The development of CVD in patients with DN, in addition to being influenced by the traditional risk factors, it is also driven by kidney-specific risk factors. Although the association between proteinuria and CVD morbidity and mortality has been demonstrated in previous studies, CVD is often under-diagnosed and under-treated in patients with DN. The present study confirms that proteinuria is involved in the pathogenesis of subclinical left ventricular systolic insufficiency in patients with DN, and that GLS was significantly lower in patients with massive albuminuria than in those with microalbuminuria. Managing lipid levels and reducing proteinuria through appropriate use of cardioprotective medications may represent a promising approach to prevent decline in cardiac function among DN patients.

Presently, the left ventricular ejection fraction (LVEF) serves as the predominant cardiac ultrasound parameter utilized for evaluating left ventricular systolic function in clinical settings. However, this research employed GLS to evaluate the comprehensive longitudinal function of the ventricle, enabling the detection of subtle changes occurring at the myocardial level. GLS has the capability to identify cardiac function abnormalities with heightened sensitivity and early detection, thereby offering more precise prognostic insights. This study was a single-center cross-sectional investigation with a relatively modest sample size, potentially leading to selection bias and a limitation in analyzing the longitudinal trajectory of cardiac function alterations. Furthermore, patients with coronary artery disease were excluded based on medical history, electrocardiogram, and echocardiogram, although the absence of coronary angiography, the gold standard for diagnosing coronary artery disease, may not have entirely ruled out asymptomatic cases. Future research could involve a longitudinal cohort study with a larger patient cohort to further assess and validate the study’s outcomes.

In conclusion, the results of this study suggest that albuminuria, hypercholesterolemia and eGFR<60 ml/min/1.73 m^2^ are strongly associated with early left ventricular systolic longitudinal myocardial dysfunction in asymptomatic diabetic patients with preserved LVEF, and that the higher cholesterol levels in patients with DN are associated with a greater risk of subclinical left ventricular systolic dysfunction.

## Data Availability

The raw data supporting the conclusions of this article will be made available by the authors, without undue reservation.
